# Asian students in the anglosphere – unravelling the unique familial pressures contributing to eating pathology: a systematic review

**DOI:** 10.1186/s40337-023-00733-y

**Published:** 2023-01-10

**Authors:** Victor Sun, Nerissa Soh, Stephen Touyz, Sarah Maguire, Phillip Aouad

**Affiliations:** 1 Westmead Clinical School, Faculty of Medicine and Health, University of Sydney, Sydney, NSW Australia; 2 Northern Clinical School, Faculty of Medicine and Health, University of Sydney, Sydney, NSW Australia; 3 InsideOut Institute, Central Clinical School, Faculty of Medicine and Health, University of Sydney, Sydney, NSW Australia

**Keywords:** Eating disorder, Disordered eating, Asian, Student, Familial, Anglosphere, CALD

## Abstract

**Background:**

There is no clear consensus on the specific familial pressures affecting Asian students in the Anglosphere, despite the validation of the Tripartite Influence model of eating disturbances in this group. However, traditional familial risk factors for disordered eating can be elevated for immigrant Asians with collectivistic-oriented familial dynamics, necessitating an examination of the culture-specific risk profile for eating pathology in student-aged Asians. This systematic review aims to consolidate and critically examine the literature on the most widely studied familial pressures related to disordered eating in Asian students in the Anglosphere.

**Methods:**

A systematic search was conducted in five databases for peer-reviewed articles measuring familial pressures and eating pathology in Asian students > 10 years old from an Anglosphere country. Following PRISMA guidelines, papers were screened by title, abstract and full text based on the eligibility criteria. Eligible studies were qualitatively analysed and synthesised narratively to assess the relationship between familial pressures and eating pathology.

**Results:**

In total, 14 papers were eligible for inclusion in the review. Eight topics related to familial stressors were identified (1) intergenerational conflict; (2) lack of familial cohesion; (3) parental overprotection; (4) low parental care; (5) familial achievement orientation; (6) parental expectations; (7) parental criticism; and (8) direct parental influence. In multiple studies, intergenerational conflict, maternal overprotection, and familial achievement orientation were significantly elevated and associated with disordered eating in US and UK Asian students, compared to white students. The studies examining parental criticism and familial cohesion had more heterogeneous findings.

**Conclusion:**

The findings demonstrate the perception of Asian parenting styles as overprotective and incompatible with individualist-oriented Western values could increase eating pathology in adolescent and university students living in Anglosphere countries. The synthesised findings of the literature also indicate disordered eating acts as a compensatory mechanism for the ongoing psychological distress generated from intergenerational conflict and familial achievement orientation. Conversely, traditional eating disorder literature on familial cohesion and low parental care may not be applicable to young Asians. Future research should focus on how social appearance anxiety and psychological factors can mediate the link between disordered eating and familial stressors in Asian students.

## Plain English summary

Family influences are known to contribute to disturbances in eating behaviours in white people and people of colour, despite cultural differences in family pressures. The Anglosphere, which describes a group of English-speaking countries with shared political and cultural heritage, has seen an increase in student-aged Asians who are vulnerable to the simultaneous pressures of Asian and Anglosphere cultures. Given this demographic is a historically underdiagnosed and undertreated group for eating disorders, this necessitates an examination of the family pressures that contribute to eating disorders which has been relatively understudied thus far. This systematic review found that cultural conflict with parents, overprotective maternal behaviours and achievement-oriented family backgrounds are consistently related to eating disturbances in Asian students in the Anglosphere. These findings also suggest that assimilation into Anglosphere culture plays a significant role in the perception of Asian family influences, and its contribution to eating pathology in this demographic. Asians in secondary and tertiary institutions internalise individual-oriented Anglosphere values through exposure to peers and media, which may conflict with community and family-oriented values of their Asian households. Continued investigation into influential factors may help inform development of culturally-sensitive guidelines for diagnosing and assessing Asian patients for eating disorders.

## Background

Eating disorder (ED) intervention studies have mainly focused on young white women, who are often stereotyped as the ‘classic ED’ patient [1]. However, shifts in research towards broad cultural associations have increasingly emphasised that people of all ethnic groups can be impacted by an ED [2]. Nonetheless, there exists a dearth of knowledge specifically on the interaction of culture-related familial pressures with ED pathogenesis [3, 4].

**Fig. 1:**
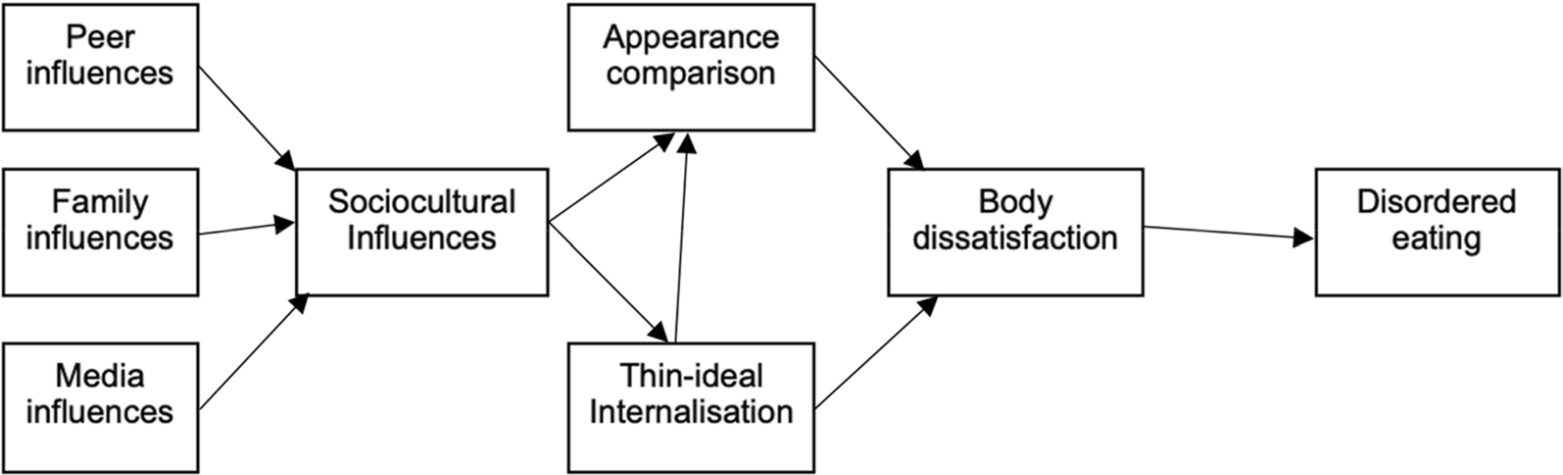
The Tripartite Influence model of body image and eating disturbance, adapted from Burke et al. [7] and Schaefer et al. [8]

Relative to other psychiatric disorders including schizophrenia and mood disorders, parental pressures are shown to be uniquely associated with EDs [5]. One of the most tested models for eating disturbance is the Tripartite Influence model, which hypothesises that familial pressures, along with peer and media influences, are integral in propagating pathological eating (Fig. 1) [6]. Although this model was first developed with predominantly white females, various studies have validated the Tripartite Influence model in men and women of colour [7, 8]. Hence, while ethnic minorities in Western countries may internalise similar peer and media influences, their culture-specific familial pressures can contribute to eating pathology and create a unique risk profile.

The Anglosphere countries of the United States (US), United Kingdom (UK), Canada, Australia, and New Zealand share common cultural and historic ties, though these nations have become more migrant-based with an influx of Asian immigrants in recent decades [9]. Although the Asian diaspora comprises diverse ethnic groups, many Asian cultures share values that distinguish them from individualist-oriented Western cultures. Asian cultures are often characterised as collectivist, meaning the needs of the family and community are prioritised over those of the individual [10]. Familial relationships across Asian cultures are characterised by a central theme of “filial piety”, a Confucian value which denotes respect, obedience and deference towards elder family members [11, 12]. Although associated with East Asian cultures, filial piety also occurs in similar themes across Indian and Arab cultures [13, 14].

Thus, familial pressures may be more influential within Asian cultures, where individuals tend to readily accept their family’s demands to avoid shame and exclusion [11]. In Anglosphere countries, racial discrimination and systemic racism represent potent challenges that Asian parents strive to protect their children from, Hence, familial pressures such as parental criticism and achievement pressures are employed by immigrant Asian parents to encourage their children’s conformity to specific behaviours which guarantee their future success and social mobility [15–17]. However, parental criticism and familial achievement orientation have classically been related to disordered eating in White-focused studies [18, 19].

The onset of EDs most commonly occur in adolescence, meaning school and university-aged Asians are a vulnerable group to eating pathology [20]. Though schools and universities are becoming more multicultural in the Anglosphere, the Asian diaspora remains an underexamined group in ED research [4]. For many young Asians living in the Anglosphere, the school environment forces them to adapt to the dominant host culture, as they are exposed to peers and the Anglosphere media [21, 22]. Recent literature has focused on racial discrimination and acculturative stress as culture-specific risk factors for disordered eating in Asian-Americans [23–26]. However, the interaction of Asian cultures, including familial stressors, with eating pathology have been underexamined in immigrant families. Consequently, there lacks a comprehensive understanding of the bicultural pressures that relate to EDs in young Asians [26], in spite of opportunities to examine the interactions of acculturation and familial pressures. As Asian students are encouraged to assimilate to Anglosphere influences on cultural values in discordance with their familial traditions, this could hypothetically cause intergenerational conflict, where the young person clashes with their parents over disagreements in cultural values and norms [27]. On the other hand, some Asian parents may engage in high parental monitoring [28], where they continually observe and direct their children’s behaviours and appearance ideals [29]. Thus, parental pressures may cause additional stress on the young person, as they are undergoing changes in social and emotional functioning, and developing their ethnic identity [30].

Despite a historic under-reporting of EDs in Asians, several studies have demonstrated Asian minorities in Anglosphere countries have a similar prevalence of ED symptoms as white people [31–33]. Additionally, ED prevalence is likely to be underestimated in the Asian diaspora, due to lower rates of ED referral and treatment-seeking compared to white people [26, 34]. However, ED treatment remains informed by studies conducted in predominantly white samples. The recognition of cultural factors, including familial stressors, that contribute to ED risk in Asian students, will be vital in providing nuanced ED assessment and treatment in this at-risk group.

Due in part to under-reporting, there is no consensus on the familial pressures that specifically contribute to eating pathology in student-aged Asian minorities in Anglosphere countries. To our knowledge, there is no systematic review thus far, that seeks to identify and critically examine the familial pressures in Asian cultures that have been related to eating pathology. A consolidation and critical analysis of the extant literature can raise cultural awareness for a fast-growing yet understudied demographic which clinicians in Anglosphere countries are increasingly assessing and managing. The contextualising of familial pressures that clinically contribute to eating disorders and the identification of future directions for research is a critical step to establish culturally sensitive guidelines that can effectively reduce the burden of familial pressures on EDs in Anglosphere Asian students.

### Aims

The objective of this systematic review is to consolidate and critically examine the findings of published studies on the most common familial stressors as they relate to disordered eating in Asian students in the Anglosphere. A secondary aim of this review is to assess the limitations and knowledge gaps in the current literature to highlight future research directions for studying familial pressures and eating pathology in young Asians.

## Methods

### Search strategy

**Fig. 2:**
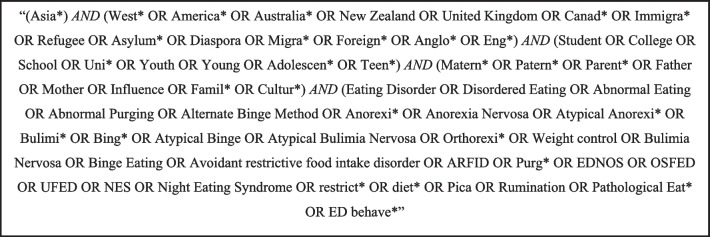
Search string used to identify relevant literature

The review followed the Preferred Reporting Items for Systematic reviews and Meta-Analysis (PRISMA) guidelines [35]. A search string (Fig. 2) containing key terms related to the population of interest, familial pressures and eating pathology was developed by two authors (V.S. and P.A.) in consultation with specific subject matter experts (N.S. and S.T.) and executed by one author (V.S.). Databases searched were Cochrane Library, Web of Science, PsychInfo, MedLine and EBSCOhost. Reverse reference list searches of eligible studies were conducted manually. The final searches were completed on 27th January 2022.

### Eligibility criteria

Studies were eligible if they were peer-reviewed with full-text availability and included at least one construct of familial pressure and at least one validated measure to assess eating pathology. The populations were restricted to Asian individuals at least ten years old, of middle-school, high-school, and university age, who reside in the US, Canada, UK, Australia or New Zealand. Asian descent was defined as self-identifying as Asian, or whose families identified as originating from Asia. Searches were limited to English-language articles. There were no restrictions on date of study and study design.

### Study selection

**Fig. 3:**
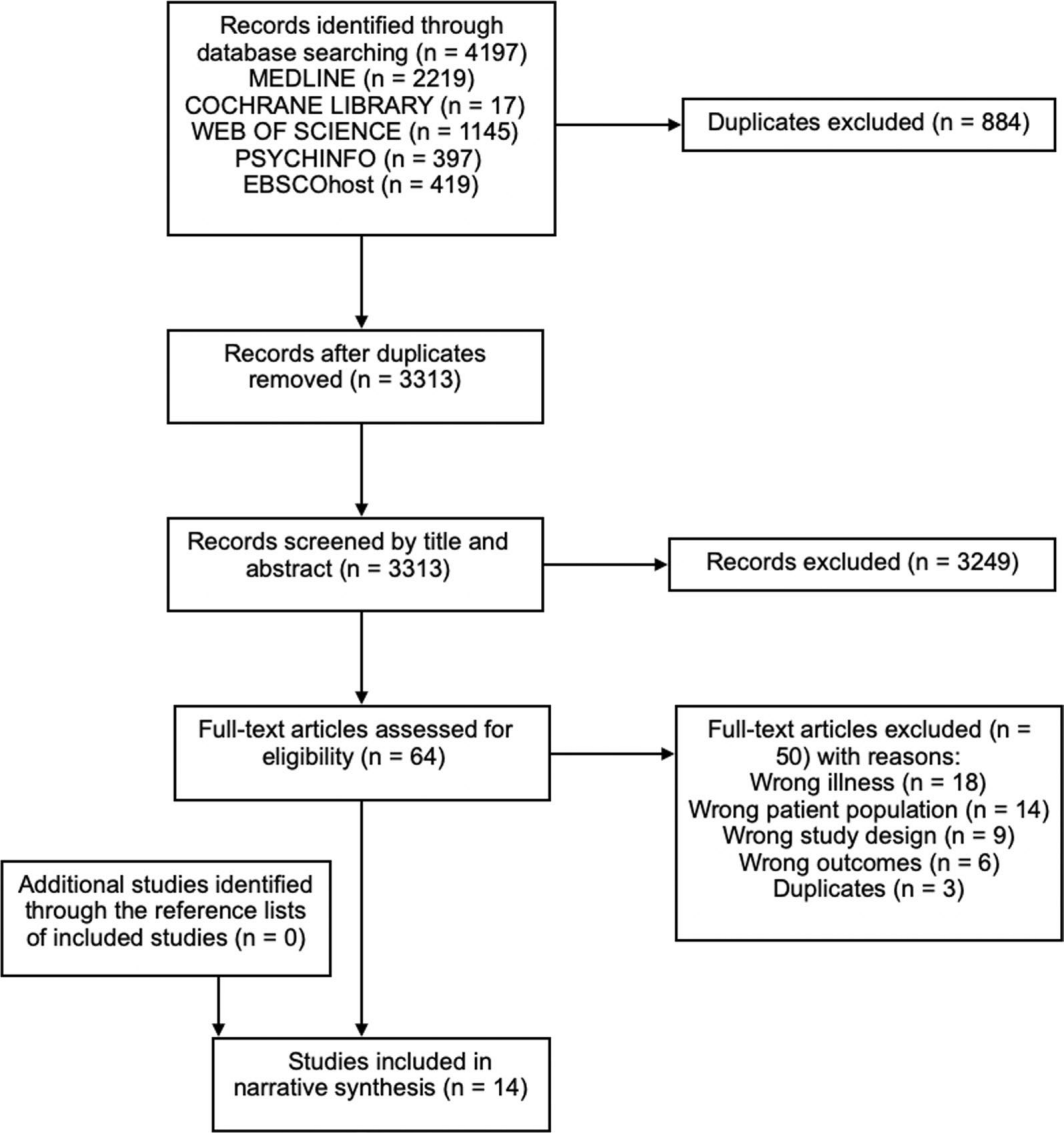
PRISMA flow diagram

The literature search identified 4197 studies from the five databases which were imported to the Covidence software platform for screening [36]. After 884 duplicates were removed, 3249 of the 3313 remaining papers were excluded after screening by title and abstract. There were 64 papers that were screened by full text, which excluded 50 studies (Fig. 3 PRISMA diagram [35]). Fourteen studies were included in the systematic review with full agreement with a second author (P.A.). The studies were critically assessed using the quality appraisal tool designed by Ferro and Speechley [37], which assessed reporting, external validity, bias and confounding, and statistical power. An inter-rater check of the short-listed articles by another author (P.A.) yielded an 80% agreement in quality assessment.

### Data extraction

The data from 14 included studies was extracted by one author (V.S.) into Microsoft Excel. Data extracted from each study focused on the aim of study, study design, education level and demographics of participants, the methodology of ethnicity determination and participant recruitment, the methodology style of familial pressures and eating pathology assessment, the measured familial pressures and eating pathology outcomes, and the relationship between familial pressures and eating pathology.

### Data synthesis

Due to the heterogeneity of the reported familial pressures and measures of eating pathology in the included studies, it was not appropriate to conduct quantitative analyses or a meta-analysis and a narrative synthesis was undertaken.

## Results

### Characteristics of included papers

The reviewed papers are summarised in Table 1. All but one of the papers were analytic cross-sectional studies, with the remaining study being a case series. Eight studies were conducted in the US and six in the UK. Four studies were conducted on undergraduate students, three on undergraduate and graduate students combined, and the remaining seven focused on middle or secondary school-aged students. Ten studies measured eating pathology through self-reported questionnaires, two used the EDE-Q interview and two examined diagnosed anorexia nervosa patients.

**Table 1:** Summary of Included studies (arranged in alphabetical order)

Author (year)	Citation	Aim	Design	Sample size	Sample characteristics	Familial stressors	Measure for family stressor	Measure for eating pathology	Findings/ results
Ahmad, Waller and Verduyn (1994)	(43)	To investigate the role of perceived parental control in development of abnormal eating attitudes in British-Asian schoolgirls	Cross-Sectional	71	British-Asian females Secondary school Mean age = 14.9 years	Parental control	PBI (Parental Bonding Instrument)	EAT-26 questionnaire	Eating pathology significantly associated with low maternal care and high maternal overprotection. Significant association between bulimia scores and maternal overprotection.
Bryant-Waugh and Lask (1991)	(40)	To examine the role of sociocultural conflict in families as a factor in emergence of eating disorders in British-Asian Anorexia Nervosa patients	Case series	4	British-Asian female Anorexia Nervosa patients 13–14 years	Parental conflict		Confirmed diagnosis of Anorexia Nervosa	Cultural conflicts between parents and children were present in all four patients.
Chang, Yu and Lin (2014)	(48)	To assess ethnic variations in perfectionism, parental and peer influences, and eating disturbances among Asian and European American females	Cross-Sectional	139	Asian-American females Tertiary education 18–23 years	Parental expectations Parental criticism Direct parental influence on eating behaviour	Frost Multidimensional Perfectionism Scale (FMPS) Family History of Eating Questionnaire	EDI-3 questionnaire	No significant association between bulimic eating, and parental expectations or parental criticism. Direct parental influence on eating behaviour did not significantly account for variance in bulimic eating.
Eisenberg et al. (2019)	(50)	To examine the association of weight-based teasing by family members with unhealthy weight control behaviours among adolescents from three immigrant communities	Cross-Sectional	480	Hmong American males and females Middle and High school	Family weight-based teasing	Yes/No survey	Questionnaire on unhealthy weight control behaviours	Hmong adolescents experienced significantly higher familial weight-based teasing than other ethnic groups. Significant association between family weight-based teasing and unhealthy weight control in combined ethnic group of girls, but not boys.
French et al. (1997)	(42)	To examine ethnic differences in psychosocial factors associated with disordered eating	Cross-Sectional	504	Asian-American females Public school Adolescents	Family connectedness	Likert-scale questionnaire of psychosocial variables	Questionnaire on dieting, purging behaviours and binge eating	Low family connectedness was a significantly associated with bingeing
Furnham and Adam-Saib (2001)	(46)	To investigate the relationship between disordered eating and perceived parental control in second-generation British-Asian schoolgirls	Cross-Sectional	122	British-Asian females Secondary school 15–17 years	Parental control	PBI	EAT-26 questionnaire	Maternal and paternal overprotection has no significant correlation with eating pathology, in combined Asian and White group
Furnham and Husain (1999)	(38)	To determine whether parental conflict and parental overprotection is associated with eating pathology in British-Asian female students	Cross-Sectional	55	British-Asian females Undergraduate Mean age = 21.15 years SD = 2.18 years	Parental conflict Parental control	Likert-scale Parental Conflict questionnaire PBI	EAT-26 questionnaire	Maternal conflict over going out, Parental conflict over choice of friends and maternal overprotection were predictors of eating pathology
Han (2020)	(30)	To examine the role of cultural and psychological factors as predictors of disordered eating among Asian and Asian American college women	Cross-Sectional	244	Asian-American females Tertiary education Mean age = 21.66 years SD = 3.35 years	Intergenerational conflict Parental attachment Family recognition through achievement	Asian-American Family Conflict Scale Experiences in Close Relationships-Relationship Structures Questionnaire Asian-American Value-Multidimensional Scale	Dutch Eating Behaviour Questionnaire (DEBQ)	Family recognition through achievement and Intergenerational Conflict are significantly associated with binge and restricted eating. Parental attachment avoidance and maternal attachment anxiety had no significant association with eating pathology.
Haudek, Rorty and Henker (1999)	(45)	To explore the associations among ethnicity, parental bonding, acculturation and eating disturbance in Asian and white American weight-concerned women	Cross-Sectional	25	Asian-American females Undergraduate Mean age = 18.8 years SD = 0.87 years	Parental control	PBI	EDE-Q interview	Asian-American women perceived mothers as significantly less caring than white-Americans. Maternal care was negatively correlated with eating concern and eating restraint.
Lee and Lock (2007)	(47)	To examine the clinical characteristics of phenomenology of anorexia nervosa in Asian-American patients in comparison to a non-Asian sample	Cross-Sectional	16	Asian-American male and female Anorexia Nervosa patients 12–18 years	Family functioning	Family Environment Scale (FES)	EDE-Q interview	Asian-American anorexia nervosa patients score significantly higher on family achievement orientation scale compared to non-Asian patients
McCourt and Waller (1995)	(44)	To examine effects of perceived parental control in eating psychopathology at diff ages in adolescence	Cross-Sectional	178	British-Asian females Secondary school 12–16 years	Parental control	PBI	EAT-26 questionnaire	British-Asians had significantly higher eating pathology than white British. Maternal overprotection significantly accounted for difference in Asians’ eating attitudes compared to white British girls, once they reached 15–16 years
Mujtaba and Furnham (2001)	(39)	To investigate if intergenerational conflict is related to eating disorder development	Cross-Sectional	118	British-Pakistani Tertiary education Mean age = 20.24 years SD = 1.78 years	Parental conflict	Likert-scale Parental Conflict questionnaire	EAT-26 questionnaire	British-Asian perceived higher parent-child conflict and eating pathology compared to the white-British and native Pakistani sample
Tomiyama and Mann (2008)	(41)	To evaluate the validity of familial enmeshment as a risk factor for eating disorders across cultural value orientations^a^	Cross-Sectional	111	Asian-American females Undergraduate	Family enmeshment	Edinburgh Family Scale (EFS)	EDE-Q questionnaire	Asian-Americans were the only racial group with no significant correlation between family enmeshment and eating pathology
Yu and Perez (2020)	(49)	To examine how maternal criticism and individual body dissatisfaction may be associated with disordered eating pathology among different cultures	Cross-Sectional	142	Asian-American males and females Undergraduate Age ≤ 23 years	Maternal criticism	Sociocultural Attitudes Towards Appearance Questionnaire (SATAQ), Likert-scale	EDE-Q questionnaire	Maternal criticism was a significant predictor of disordered eating in Asian-Americans, more saliently linked than White and Latinx group

^a^Familial Enmeshment refers to familial relationships defined by high levels of overprotectiveness and lack of personal boundaries.

The mean score of the Quality Index was 8.21. The case series had the lowest score of 3, while the scores for the cross-sectional studies ranged from 7 to 12. The domains that contributed most to poor quality were lack of power calculation, inadequate adjustment for confounders and unrepresentative population, as most studies excluded men.

The key findings have been organised based on eight familial themes that were identified across the 14 studies, and the relationship with eating pathology is outlined for each theme.

#### Intergenerational conflict

Intergenerational conflict refers to friction between parents and child due to an acculturation mismatch which leads to dissonance in cultural values and communication [27].

Two studies demonstrated a significant association between intergenerational conflict and disordered eating [30, 38]. Mujtaba and Furnham specifically found that maternal conflict over going out and parental conflict over choice of friends were the dimensions of intergenerational conflict that were significantly associated with unhealthy eating attitudes in British-Asian undergraduate females [38]. Han found the increased frequency and seriousness of intergenerational conflict was linked with restricted and binge eating [30]. Mujtaba and Furnham found British-Pakistani university students had significantly higher familial conflict compared to white British and native Pakistani students [39]. The case series conducted by Bryant-Waugh and Lask reported cultural conflict was experienced by all four British-Asian anorexia patients [40].

#### Lack of familial cohesion

Familial cohesion refers to the level of emotional bonding and autonomy in relationships between parents and children. Familial relationships which lack cohesion can range from enmeshment signalling interactions which lack boundaries, to low family connectedness. Tomiyama and Mann [41] found Asian-Americans were the only ethnic group to have a non-significant relationship between familial enmeshment and ED pathology. Similarly, Han [30] found no significant association between insecure parental attachment and restricted eating. In contrast, French et al. [42] demonstrated binge eating and low family connectedness had a significant association in Asian-American adolescents.

#### Parental overprotection

Parental overprotection or control encompasses excessive restriction of a child’s autonomy. Four studies found a significant association between maternal overprotection and disordered eating [38, 43–45]. McCourt and Waller [44] found maternal overprotection was significantly associated with disordered eating attitudes and behaviours in British-Asian girls, once they reached 15–16 years. Paternal overprotection was correlated in the latter study with eating pathology [44] and significantly associated with eating concern in the study conducted by Haudek, Rorty and Henker [45]. However, Ahmad, Waller and Verduyn found the relationship between paternal overprotection and eating pathology was non-significant [43]. Two studies also demonstrated the group of white school-aged girls had no significant association between increased parental control and maladaptive eating attitudes [43, 45].

#### Low parental care

Parental care is defined as an individual’s perception of warmth and nurturance from their parents. Low maternal care was significantly correlated with eating restraint and concern in two papers [43, 45] although this was unsupported by Furnham and Husain [46]. Furnham and Husain found that low paternal care was significantly correlated with eating pathology [38], while two papers found no significant correlation between the two [43, 45].

#### Familial achievement orientation

Achievement-oriented families are defined as those that promote their children’s obligations for academic success and accomplishments to appease their family’s desires. In an ethnically diverse sample, Lee and Lock [47] found families of Asian AN patients valued achievement significantly more higher than non-Asian patients. Han [30] determined that familial achievement orientation was significantly associated with binge and restricted eating.

#### Parental expectations

Chang et al. [48] found parental expectations of perfectionism had no correlation with bulimic behaviour in an Asian-American college sample.

#### Parental criticism

Three papers found Asian-Americans experienced the most parental criticism of all ethnic groups [48–50]. Maternal criticism was the strongest predictor of disordered eating in the Asian-American female undergraduates studied by Yu and Perez [49], while Chang et al. [48] showed no significant association between parental criticism and bulimic behaviours.

#### Direct parental influence

Direct parental influence differs from parental criticism, in referring to parents directly pressuring their children towards dieting and weight loss. Chang et al. [48] found direct parental influence on eating behaviour and weight control was significantly associated with bulimic behaviour in Asian-American female university students.

## Discussion

The extant literature indicated this was the first study to systematically review, consolidate and critically examine familial stressors in Asian students living in Anglosphere countries. This systematic review critically examined the evidence for relationships between familial stressors and disordered eating, in Asian students from Anglosphere countries. Eight themes in familial pressures were identified across the 14 included studies including (1) intergenerational conflict; (2) lack of familial cohesion; (3) parental overprotection; (4) low parental care; (5) achievement orientation; (6) parental criticism; (7) parental expectations; and (8) direct parental influence. The results indicated that intergenerational conflict, maternal overprotection, and familial achievement orientation were the most noted familial pressures significantly associated with eating pathology in this population.

### Intergenerational conflict

Intergenerational conflict over cultural values is a distinct familial pressure encountered by Asian students in the Anglosphere, as their adoption of Western values of assertiveness and individual desire comes into conflict with Asian cultural values of filial piety and collectivism [39]. The findings of this review aligned with a meta-analysis which found the conflicting demands of Anglosphere and non-Western cultures creates an acculturation mismatch between traditional parents and acculturated children, which correlates with intergenerational cultural conflict [51]. The same meta-analysis found intergenerational conflict worsened as immigrant children aged and increasingly assimilated, which could highlight why cultural conflicts are still potent for university subsamples. Similarly, Mumford et al. demonstrated that the traditional orientation of parents was significantly associated with higher eating attitudes test (EAT) scores in British-Asian girls [52], in alignment with the review’s findings. Given that a greater acculturation gap is predictive of intergenerational conflict, this can leave immigrant Asian children from traditional families vulnerable to developing disordered eating, particularly when they reach adolescence and begin to internalise the derogatory stereotyping of Asians in Western culture which encourages them to separate from their family’s culture and assimilate into the white mainstream [52–54]. The resentment and emotional distress that Asian youth experience from conflict with parents who fundamentally do not understand their children’s desire to individuate and assimilate, has additionally been linked by Asian patients to ED development as a coping mechanism [55]. Thus, intergenerational conflicts can serve as a continual stressor that maintains EDs in Asian patients, which are challenging to address without culturally appropriate interventions [53].

### Lack of familial cohesion

Dysfunctional familial cohesion is known to influence the development and maintenance of eating pathology, as families of ED patients have been commonly characterised as having high enmeshment and a lack of familial cohesion [56, 57]. However, the findings of this review have exhibited an opposing view [30, 41], where familial enmeshment and insecure parental attachment has not been pathological for Asian youth. Kirschner [58] found familial enmeshment may be more culturally normative in collectivist Asian cultures, than for independence-oriented Western cultures where such interactions can increase psychological stress. These findings align with a study in Korean-American families by Jin and Roopnarine, which hypothesised that enmeshment may not be pathological in collectivistic-oriented families that embrace close proximity in family relationships as culturally normal [59]. Familial enmeshment may be perceived by Asians as signalling unconditional commitment to well-being whilst white individuals culturally perceive the negative aspects of enmeshment including the suffocating lack of boundaries and over-dependence on family [60]. In contrast, low family connectedness does not align with the family-oriented values of Asian cultures, which could underlie its significant association with pathological eating in Asian-American students in French et al. [42]. However findings by Soh, Touyz and Surgenor found a conflicting overview of the relationship between familial cohesion and disordered eating in Asians [58, 61]. The latter review hypothesised that the level of satisfaction with the perceived family environment could be linked to more disordered eating [61]. Thus, cultural differences that influence a patient’s perception of their family relationships could explain why Asian students do not significantly exhibit eating disturbances in response to traditional ED risk factors like familial enmeshment and insecure attachment [30, 41].

### Parental overprotection

Parental overprotection is common in Asian family dynamics, for parents to express care and support to their children’s development [62], and can extend into adulthood for young Asians [28]. In the context of Anglosphere countries, Asian students may conversely believe that their non-Asian peers are often offered more agency and independence. Consequently, this parenting style has been suggested to be maladaptive to young Asians who grow up internalising the Anglosphere’s values of autonomy and individual freedom [63]. While traditional Asian parents may believe increased parental monitoring is important in preparing their children for success in predominantly white environments [62, 64], this may foster resentment and psychological distress in their adult children who perceive this parenting behaviour as abnormally intrusive in the context of Anglosphere culture [28, 65]. The link between maternal overprotection and eating disturbances could be explained by an Asian child’s attempt at claiming control of their lives [38, 44] or to compensate for psychological distress [65]. However, adolescents with overprotective parents may suppress their need for autonomy to avoid intergenerational conflict, which has been related to emotional dysregulation and psychological risk factors [66, 67]. Asian mothers tend to be protective and highly involved in their children’s daily activities, especially in the upbringing of their daughters [17]. This could explain why maternal control was linked to disordered eating in all the included studies that examined this familial pressure, while paternal control has a more conflicting relationship. It is important to note that no paper that assessed parental control included males, and Asian men typically view their fathers as more controlling and influential than Asian women [17].

### Low parental care

In classical ED literature, bulimia and anorexia nervosa patients have commonly reported low parental care [68, 69], which is traditionally mediated by internalised shame [70]. Asian students are generally raised by authoritarian and low-warmth parenting [71], in contrast to Western cultures where parental love is equated with high parental care [45]. The literature has typically described parental care as a protective factor against health-risk behaviours in white children, including eating pathology [72, 73]. However, in Asian cultures, parental care is typically expressed indirectly through acts of services and parental sacrifice, rather than through warm parenting [74]. Furthermore, the media stereotyping of Asian parents as controlling and emotionally unavailable, can further contribute to internalised shame in acculturated Asians, and lead to entrenched ideas of the specific behaviours that constitute warm parenting [62].

Hahm et al. [72] hypothesised that highly acculturated Asian adolescents may feel separated from their family’s cultural values, leading to a failure to accept low-warmth parenting as their peers with more traditional cultural orientations. Conversely, a study of Chinese-Australian women found high parental care was positively correlated with eating pathology in traditional women, in contrast with previous findings of its protective value [75]. Overall, the extant literature seems to align with the conflicted findings on the relationship between low parental care and eating pathology in Asians in the Anglosphere. Drawing on the mediating factors of internalised shame may explain the heterogeneity of findings, where low parental care may manifest in psychological distress and eating pathology when its perception is abnormal. In contrast, the traditional notion of low parental warmth is shown to manifest differently in Asians who perceive such parenting styles as culturally normative, and equate their parent’s behaviour with emotional care.

### Family achievement orientation

Traditional literature on white families have supported higher levels of achievement orientation in the family environments of individuals with anorexia nervosa or binge eating disorder [18, 76], though Lee and Lock found Asian anorexia patients had a significantly higher frequency of an achievement-oriented family background [47]. Academic success is viewed commonly by immigrant Asian families as the only guaranteed pathway for their children to be shielded from poverty and racial discrimination in white-dominant societies [77]. Hence, Asian parents strive to secure their children’s upward mobility in Anglosphere countries by installing a commitment to school and life success, but these achievement pressures have been linked to perfectionistic strivings and emotional distress [78]. The findings of this systematic review are supported by a qualitative study conducted by Smart et al. [55] where ED therapists reported their female Asian-American patients were significantly impacted by their desire to appease parents with their achievement. Smart et al. affirmed that female Asian-American patients were inclined to meet their parent’s standards on appearance to preserve familial pride and avoid shame [55]. The failure to meet parental standards on appearance, can manifest in shame for young Asians. Asian women with ED were described as motivated by an avoidance of shame, to prevent themselves from disappointing their families and continue striving for ideals of thinness [55].

Other studies have hypothesised that the emotional distress created from heightened achievement pressures in Asian families may drive young girls to compensate through disordered eating [30, 79]. The perceived social pressures may carry more weight for female Asian students, who become predisposed to developing pathological eating habits to preserve familial pride and strive for an ideal appearance.

### Parental expectations

Multiple studies have demonstrated that Asians report higher levels of perfectionism than white people [48, 80, 81], which has been linked to authoritarian parenting styles and higher parental expectations in Asian families [15, 82]. The findings of this systematic review conflicted with other Asian-American studies, which demonstrated a correlation between high parental expectations and disordered eating in participants [81, 83]. However, a more recent study by Brosoft, Calebs and Levinson found that although perfectionism was higher in Asian-Americans, it was more strongly associated with ED symptoms in European-Americans [80]. This could explain why perfectionism has been traditionally linked with eating pathology. The same study by Brosoft, Calebs and Levinson demonstrated that social appearance anxiety was predictive of ED symptoms in Asian-American undergraduates.

Goel et al. found a focus group of South Asian-American women internalized high parental expectations for appearance, in addition to academic success [84]. These heightened parental expectations of appearance ideals were linked with body dissatisfaction, a significant risk factor for eating disturbances as shown in Fig. 1 [85]. Unrealistic parental expectations of body image may be internalised by Asian children, who are raised in a culture of filial piety to become obliged to desire familial approval [55, 84]. This is further concerning in Anglosphere countries, where Asian students internalise Eurocentric beauty standards from Anglosphere media and peer influences, which can be inconsistent with familial expectations of appearance [86]. A study of Asian-American women suggested that bicultural stresses contribute to higher levels of body dissatisfaction and disordered eating, while pressure from Western or Asian cultures alone may not increase risk [78]. Similarly, a qualitative study on Asian-American women reported most participants felt their body image was negatively impacted by their limitations to achieve Eurocentric beauty standards and the pressure to conform to familial expectations of attractiveness [86]. This conflict in beauty ideals may fuel a constant dissatisfaction with one’s body shape and weight, driving pathological eating behaviours in Asian students experiencing high parental expectations in the context of bicultural pressures.

### Parental criticism and direct parental influence

Parental criticism has traditionally been correlated with disordered eating in the literature [19], particularly with bulimia [87, 88]. In Asian cultures, parental criticism is culturally normative where it is employed in families to promote behaviour change in children [84, 89]. This systematic review observed conflicting findings on the association of parental criticism with eating pathology. Yu and Perez focused specifically on maternal criticism of appearance and weight, demonstrating a significant association with disordered eating. This aligned with previous studies where active parental criticism centred on body shape or size were predictive of disordered eating [19, 90], along with studies indicating maternal pressures are more potent in influencing eating behaviours [90, 91]. Similarly, Chang, Yu and Lin, also identified by this systematic review, found parental criticism framed as a direct influence on dieting and weight loss was significantly associated with eating pathology in Asian-American college students [48].

Culturally, blunt interactions in Asian families have been related to enforcing weight standards and expressing judgement on a child’s eating behaviours [50, 84]. Also, there are Asian parental beliefs that children have better chances of success in an Anglosphere country when conforming to Western ideals of beauty [89]. Asian parents intend to use criticism to exhibit their care and to shield their children from further shame [92], but immigrant Asian students may associate increased parental criticism with Western-influenced connotations of being unloved and inadequate, rather than the parents’ intentions of expressing support and care [93]. Kawamura found that the contribution of parental criticism to body dissatisfaction, was mediated by whether Asian children perceived these comments as supportive or overcritical [92]. This hypothesis has been supported by qualitative studies, where Asian-American women reported interpreting parental criticisms about appearance as hurtful and directly related to negative body image and eating concerns [79, 93]. That parental comments carry more weight for Anglosphere Asian students [15], combined with parental criticism in Asian families being culturally normative, may render parental criticism a potentially potent risk factor for disordered eating in the Anglosphere Asian diaspora.

### Limitations and strengths

This review has several limitations. Almost all included studies were cross-sectional and could not demonstrate whether familial pressures cause eating pathology. The review covered students of Asian descent, but Asia is highly culturally and ethnically diverse. Future studies could focus on intra-Asian differences in factors that determine disordered eating. The review excluded American studies that combined Pacific Islanders with the Asian-American group as the focus was on Anglosphere students of Asian descent, although this led to the exclusion of studies that examined the relationship between familial stressors and eating pathology of Asian participants.

The studies included in this systematic review encompassed Asian participants of all generational status, though the level of acculturation differs between Asian families headed by first and third-generation immigrants. Hence, the findings may be less applicable to Asian students raised by Westernised parents that lack collectivist orientation.

The quality of studies was another limitation, as most studies did not calculate statistical power or adjust for confounding. Furthermore, the exclusion of men in 11 of the studies, meant most included studies were unrepresentative of the general population of Asian students.

Eligible studies were from the UK and US, and none were identified from Australia, Canada, or New Zealand. This reduces the scope of the review’s coverage of Asian students in the Anglosphere. As the included studies were conducted in students, this could reduce the generalisability to young Asians not enrolled in studies.

This review’s methodology was also skewed towards studies that examined the contribution of familial pressures to eating pathology as opposed to protective factors for healthy eating behaviours in student-aged Asians in Anglosphere countries.

The strengths of the current review include the extensive range of familial pressures that were critically examined in the context of eating pathology in acculturating Asian students. The review consolidates 30 years of research to comprehensively explore the intersection of culture and disordered eating to address the dearth in literature regarding the bicultural pressures faced by Asians in Anglosphere countries. This review contextualises the familial pressures which could potentially contribute to eating pathology in this understudied demographic, and can facilitate for clinicians to consider cultural factors when treating Asian patients in Anglosphere countries. The included studies encompass a broad age range which has greater applicability to Asian adolescents enrolled in secondary and tertiary education when compared to studies focused on a narrow age category. Another strength is the validity and reliability of outcome measures in all cross-sectional studies.

### Future directions and implications

Future intervention studies should test whether addressing mediating psychological factors, such as internalised shame and social appearance anxiety, can mitigate the association of specific familial pressures and eating pathology in young Asians. The cultural nuances of parent-child relationships in immigrant Asian families require additional examination, specifically in exploring how perceptions of familial cohesion, low parental care and gender comparisons in parental control are related to eating pathology. Future studies should also focus on the relationship between familial pressures and eating pathology in Asian students living in Canada, Australia and New Zealand, as the extant literature on Anglosphere Asian students is limited to the United States and United Kingdom. Future studies may wish to further examine the influences of racial discrimination and internalised racism on eating pathology and familial pressures in the Anglosphere Asian diaspora.

Finally, future studies should address quality issues in the existing literature by increasing Asian male representation, adjusting for confounders and calculating statistical power.

The findings of this review can provide a foundational understanding of culturally-specific familial stressors, to enable future studies to address the dearth of knowledge in treatment of Asian patients [94]. The practice of culturally-sensitive ED assessment can mitigate the historic underscreening and underdiagnosis of Asian ED patients, while lowering barriers for patients concerned about clinician discrimination or misunderstanding [1].

## Conclusion

Overall, this review affirms that collectivist-oriented Asian families foster a unique risk profile for eating pathology in student-aged Asians in the Anglosphere. Ultimately, intergenerational conflict, maternal overprotection, and familial achievement orientation warrant further examination as the culture-specific pressures that are elevated in Asian family systems and linked with eating pathology. Further examination of the culture-specific familial pressures faced by Asian youth and other ethnic minorities, will be critical to informing clinical guidelines for assessing and treating pathological eating in a multicultural context.

## Data Availability

Not applicable. Data requests for specific eligible studies mentioned in this review are at the discretion of the data owner and/or participating institutions.

## Abbreviations

EAT: Eating attitudes test
ED: Eating disorder
PRISMA: Preferred Reporting Items for Systematic Reviews and Meta Analyses
